# Extending heart preservation to 24 h with normothermic perfusion

**DOI:** 10.3389/fcvm.2024.1325169

**Published:** 2024-04-04

**Authors:** Brianna L. Spencer, Spencer K. Wilhelm, Christopher Stephan, Kristopher A. Urrea, Daniela Pelaez Palacio, Robert H. Bartlett, Daniel H. Drake, Alvaro Rojas-Pena

**Affiliations:** ^1^ Extracorporeal Life Support Laboratory, Department of Surgery, University of Michigan Medical School, Ann Arbor, MI, United States; ^2^ Department of Cardiac Surgery, University of Michigan Medical School, Ann Arbor, MI, United States; ^3^ Department of Surgery, Section of Transplantation, University of Michigan Medical School, Ann Arbor, MI, United States

**Keywords:** heart preservation, normothermic, *ex situ*, *ex vivo*, prolonged, perfusion, extracorporeal support

## Abstract

Cold static storage (CSS) for up to 6 h is the gold standard in heart preservation. Although some hearts stored over 6 h have been transplanted, longer CSS times have increased posttransplant morbimortality. Transmedics® Organ Care System (OCS™) is the only FDA-approved commercial system that provides an alternative to CSS using normothermic *ex situ* heart perfusion (NEHP) in resting mode with aortic perfusion (Langendorff method). However, it is also limited to 6 h and lacks an objective assessment of cardiac function. Developing a system that can perfuse hearts under NEHP conditions for >24 h can facilitate organ rehabilitation, expansion of the donor pool, and objective functional evaluation. The Extracorporeal Life Support Laboratory at the University of Michigan has worked to prolong NEHP to >24 h with an objective assessment of heart viability during NEHP. An NEHP system was developed for aortic (Langendorff) perfusion using a blood-derived perfusate (leukocyte/thrombocyte-depleted blood). Porcine hearts (*n* = 42) of different sizes (6–55 kg) were divided into five groups and studied during 24 h NEHP with various interventions in three piglets (small-size) heart groups: (1) Control NEHP without interventions (*n* = 15); (2) NEHP + plasma exchange (*n* = 5); (3) NEHP + hemofiltration (*n* = 10) and two adult-size (juvenile pigs) heart groups (to demonstrate the support of larger hearts); (4) NEHP + hemofiltration (*n* = 5); and (5) NEHP with intermittent left atrial (iLA) perfusion (*n* = 7). All hearts with NEHP + interventions (*n* = 27) were successfully perfused for 24 h, whereas 14 (93.3%) control hearts failed between 10 and 21 h, and 1 control heart (6.6%) lasted 24 h. Hearts in the piglet hemofiltration and plasma exchange groups performed better than those in the control group. The larger hearts in the iLA perfusion group (*n* = 7) allowed for real-time heart functional assessment and remained stable throughout the 24 h of NEHP. These results demonstrate that heart preservation for 24 h is feasible with our NEHP perfusion technique. Increasing the preservation period beyond 24 h, infection control, and nutritional support all need optimization. This proves the concept that NEHP has the potential to increase the organ pool by (1) considering previously discarded hearts; (2) performing an objective assessment of heart function; (3) increasing the donor/recipient distance; and (4) developing heart-specific perfusion therapies.

## Introduction

Cold static storage (CSS) for up to 6 h has historically been the gold standard in heart preservation. Although some hearts stored over 6 h have been transplanted, longer CSS times have increased posttransplant morbimortality secondary to cold ischemia, endothelial damage, and loss of vasomotor tone. Recently, there has been increased research on normothermic *ex situ* heart perfusion (NEHP) for prolonged storage of heart tissues prior to transplantation.

Transmedics Organ Care System (OCS™) is the only FDA-approved commercial perfusion system that provides an alternative to CSS using NEHP in resting mode with aortic perfusion at the rate of coronary perfusion (Langendorff method). However, it is also limited to 6 h and lacks an objective assessment of cardiac function.

Over the past 7 years, the Extracorporeal Life Support Laboratory at the University of Michigan has worked to prolong NEHP. We demonstrated that hearts could be routinely perfused and preserved for 3 days when an aliquot of plasma was continuously exchanged between the blood perfusate and a live paracorporeal animal (sheep). Something is removed and/or added that allows long-term successful NEHP. These factors are humoral (not cellular) and present in plasma (1). We conducted a series of experiments to identify those factors without the paracorporeal animal. This was done in a stepwise fashion beginning with fresh donor plasma exchange for 24 h in piglet (small-size) hearts (2). These experiments demonstrated that the critical factors are added/removed by plasma exchange alone. Next, we evaluated hemofiltration to assess the toxic factor removal component of plasma exchange. Hemofiltration at 1 cc/g/h allowed successful 24-h perfusion. We evaluated the filtrate to determine which critical molecules are removed. The only nutrition in this series of studies was glucose added in the filtration replacement fluid (3). All of these studies were done with an aortic perfusion of the coronary circulation at around 1 mL/min/g of cardiac tissue. Studies are ongoing to evaluate left atrial (working mode) perfusion to measure heart function during NEHP (publication pending). In this report, we summarize our published and ongoing experience with NEHP without a paracorporeal animal.

## Methods

### Animals

Forty-two healthy pigs (6–55 kg) were utilized during 24 h NEHP runs with various interventions. Piglet (small-size) hearts: (1) Control NEHP without interventions (*n* = 15); (2) NEHP + plasma exchange (*n* = 5); (3) NEHP + hemofiltration (*n* = 10). Juvenile pig (adult-size) hearts were used to prove the adequate support of larger hearts NEHP + hemofiltration (*n* = 5) and the assessment of heart function in real time while on NEHP with intermittent left atrial (iLA) perfusion (*n* = 7). All animals received humane care in accordance with the National Institutes of Health Guide for the Care and Use of Laboratory Animals, and protocols were approved by the University of Michigan Institutional Animal Care and Use Committee (Protocol # 11170, Approved 18 January 2023).

### Surgical procedure

The surgical procedure was previously described by Tchouta et al. (2) and Johnson et al. (3) and is unchanged from prior experiments. A brief description is as follows. Isoflurane-inhaled general anesthesia was induced with ketamine–zolazepam (7 mg/kg) combined with xylazine (3 mg/kg). The skin was prepared and draped in a standard sterile manner, and intravenous antibiotics were administered (nafcillin 25 mg/kg and gentamicin 2.25 mg/kg). Lidocaine (1 mg/kg) was administered intravenously before midline sternotomy. The pericardium was left intact to minimize tissue desiccation. The extrapericardial great vessels were isolated and loosely encircled with ligatures. The animals received intravenous unfractionated heparin 400 IU/kg (Sagent Pharma, Schaumburg, IL, USA). Following documentation of adequate systemic anticoagulation, a cardioplegia needle was placed in the proximal innominate artery and the distal innominate and left subclavian arteries were ligated. The proximal intrathoracic inferior vena cava (IVC) and the left azygous vein were ligated, and the pig was exsanguinated through the distal IVC using a 20–24Fr cannula and standard sterile blood collection bags. Concomitantly, the mid-descending thoracic aorta was cross-clamped, the superior vena cava (SVC) was ligated, and 1 L of cold (5 °C) del Nido cardioplegia (CAPS Inc., Detroit, MI, USA) was subsequently administered through the proximal innominate artery. The left heart was decompressed by transecting the right pulmonary veins. Sterile saline ice slush was applied to the heart during cardioplegia administration. After the administration of cardioplegia and confirmation of cessation of heart function, the hearts were excised with the pericardium intact, weighed, and placed in an ice bath for back table preparation aiming for <60 min of cold ischemia time (CIT).

### Back table preparation

A 10Fr venous drainage cannula (Medtronic, Minneapolis, MN, USA) and a customized high-compliance balloon, secured to the cannula (Dynarex, Orangeburg, NY, USA), connected to a pressure transducer apparatus, were inserted into the left ventricle (LV) to monitor LV pressure. A 5-0 polypropylene suture was placed into the posterior leaflet of the mitral valve and secured to the cannula to prevent balloon migration. The modified LV compliance balloon was used for all NEHP studies except for the ones where iLA perfusion was performed, as LV ejection was required. A 20Fr DLP venous drainage cannula (Medtronic Inc., Dublin, Ireland) was placed into the right ventricle (RV) via the pulmonary artery (PA). A customized 1/4 × 3/8-in connector with Luer Lock (Medtronic, Minneapolis, MN, USA) was secured in the aortic arch for antegrade coronary perfusion (Langendorff). All remaining branches were ligated, including the SVC, IVC, subclavian artery, and innominate artery. The heart and cannulae were de-aired and connected to the NEHP system.

### Circuit, circuit priming, and blood-derived perfusate

**Figure 1:**
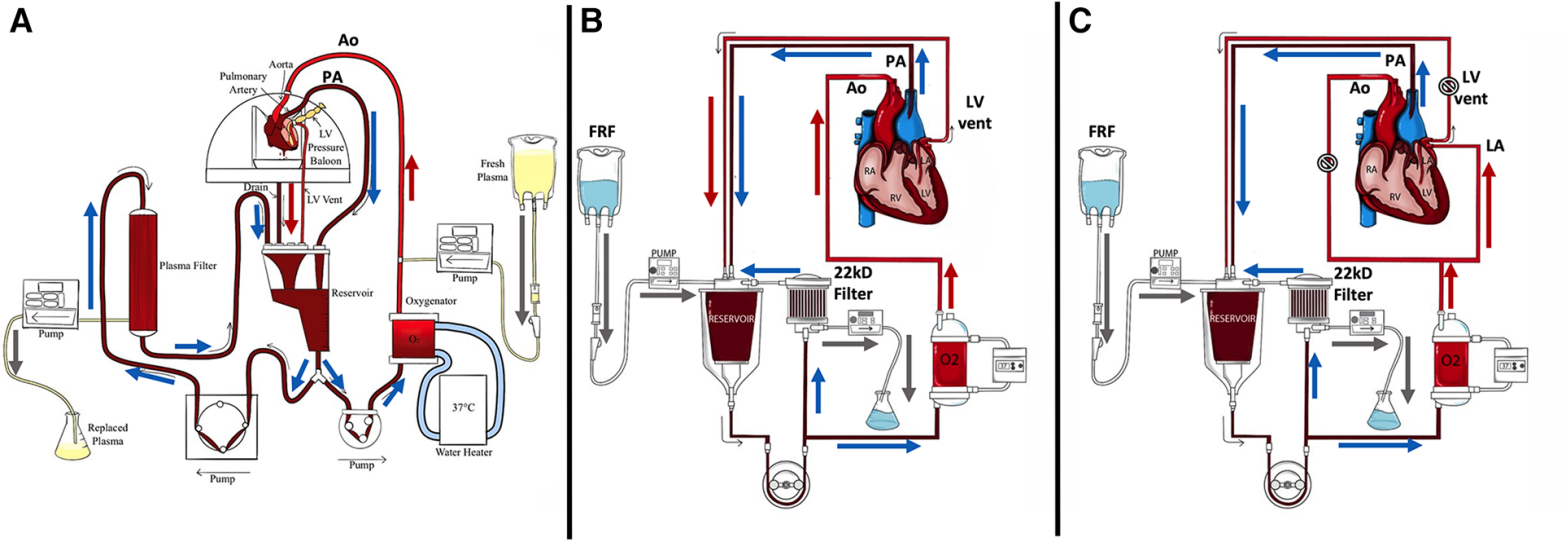
Diagrams of NEHP circuits. (**A**) An NEHP circuit used for pediatric hearts with plasma exchange (piglet hearts) using a plasma separator (Plasmaflo OP-05W[A] (Asahi Kasei Medical MT Corp., Oita, Japan); (**B**) An NEHP circuit used for pediatric (piglet) and adult (juvenile pigs) heart models with hemofiltration [Prismaflex HF1000 filter (22Kd) Baxter Inc., Deerfield, IL, USA]; and (**C**) NEHP circuit modifications for adult (juvenile pigs) heart iLA perfusion studies. Ao, aortic root line; FRF: Filtrate Replacement Fluid; LV: Left Ventricle; PA: Pulmonary Artery line. 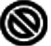, clamping of line for iLA perfusion.

The perfusion circuit (Figure 1) consisted of a reservoir (Terumo, Ann Arbor, MI, USA), an FX05 Baby Capiox Oxygenator (Terumo CVS, Ann Arbor, MI, USA), and an Mpump (peristaltic-modified roller pump) (Daris LLC, Ann Arbor, MI, USA). The priming of the circuit contained several steps: (1) 400–500 mL of Plasma-lyte A (balanced crystalloid solution) to remove any air in the system; (2) Plasma-lyte A is removed from the system (as much as possible); and (3) addition of a blood-derived perfusate from the IV transfer bag (see below) into the NEHP reservoir.

Under general anesthesia, healthy pigs (100–120 kg) were exsanguinated and used as blood donors. Blood (5–6 L) was collected using the three-bag Teruflex® (Terumo Corp., Tokyo, Japan) blood bag system with citrate phosphate dextrose adenine solution (CPDA-1) anticoagulant. The volume of blood per kit was 450 mL. The collected blood was then stored in a 5 °C refrigerator for up to 10 days on a rocking system until its use. Whole blood was then separated using the collecting blood bags via centrifugation (Sorvall Legend XFR Centrifuge—Thermo Fisher Scientific, Waltham, MA, USA) for 20 min at 25 °C with 3,600 RPM. Plasma and packed red blood cells (pRBCs) were then collected using the plasma extractor and Fenwal transfer set (Fresenius Kabi AG, Bad Homburg, Germany), and the buffy coat (platelet and white blood cells) was discarded.

The priming volume was approximately 250–300 mL of platelet- and leukocyte-reduced blood with a hemoglobin (Hb) concentration goal >8 g/dL and hematocrit ≥24% for all studies. The blood-derived perfusate was then oxygenated and conditioned to normothermic conditions (37 °C) prior to connecting the heart to NEHP. Washed pRBCs from our animal blood bank were used to maintain Hb >8 g/dL. Normal saline was used to wash pRBCs if the K values were >9 mmol/L. In addition, calcium was monitored immediately after pRBC and hourly during NEHP and replenished if ionized Ca <1.1 mmol/L with 250 mg calcium gluconate (1 cc bolus). Glucose was replaced if perfusate levels were <60 mg/dL, but this situation rarely occurs during NEHP with the addition of plasma exchange or hemofiltration, unless contamination with bacteria is observed.

### Perfusion protocol

Aortic blood flows were slowly increased and adjusted to maintain coronary blood flow between 0.5 and 1.0 mL/min/g of cardiac tissue (mL/min/g) with a mean flow of 0.7 mL/min/g concordant with physiologic coronary blood flow using Langendorff perfusion. PA outflow and LV drainage were collected and returned to the reservoir of the perfusion circuit. The LV compliance balloon was inflated to maintain initial end-diastolic pressure within 8–10 mmHg; the initial volume required to reach this pressure was kept consistent throughout the duration of the prep. PA venous saturations were targeted to 75%–90%, and coronary flow adjusted accordingly. The temperature was maintained at 37 °C using a water heater (CSZ Cincinnati Sub-Zero ECMO Heather, Cincinnati, OH, USA). The sweep gas (50% O_2_, 45% N_2_, and 5% CO_2_) was adjusted to maintain pCO_2_ at 40 ± 5 mmHg. If fibrillation occurred, the heart was defibrillated with 5–10 J using internal defibrillation paddles (Philips, Andover, MA, USA). The perfusate was exchanged at 60 min of NEHP to eliminate residual cardioplegia and toxins that may have accumulated from reperfusion. The perfusate exchange followed cardiopulmonary bypass practices. The volume in the reservoir was depleted to a safe level (−30 cc), and the new perfusate was added while the outflow from the heart was collected and discarded. This maneuver mitigated air embolism, while most of the perfusate was exchanged without a stoppage of perfusion of the heart.

## Summary of individual experiments

### Plasma exchange

Plasma was infused into the aortic catheter in the experimental hearts and filtered out at a similar rate from the reservoir drainage line. The previous plasma cross-circulation experiments included removal of plasma from the perfusion circuit and return to the paracorporeal animal, creating continuous plasma exchange (2). In the current experiments, continuous plasma exchange was done by a continuous infusion of bank plasma and continuous removal of the same amount of plasma by a plasma separator [Plasmaflo OP-05W(A), Asahi Kasei Medical MT Corp., Oita, Japan] (Figure 1A).

### Hemofiltration

Perfusion was performed in parallel with hemofiltration (3). The hemofilter was Prismaflex HF1000 (Baxter Inc., Deerfield, IL, USA), which filters molecules up to 22 kD. Perfusate hemofiltration was maintained at 1 mL/h/g using an IV pump. Isotonic filtrate replacement fluid (FRF) was added to the perfusate at a 1:1 ratio. One liter of FRF consisted of 750 mL 0.9% saline solution and 250 mL with 3.3 g of glucose, 400 mg calcium gluconate, 30 mEq bicarbonate, 160 mg magnesium, 4 mEq potassium, 250 mg nafcillin, and 40 mg of gentamicin (Figure 1B).

### iLA perfusion

For the iLA perfusion, a 10Fr venous drainage cannula was inserted into the LV for LV drainage. A 20Fr DLP venous drainage cannula (Medtronic Inc., Dublin, Ireland) was placed into the RV via the PA. A 1/4 × 3/8-in connector with Luer Lock (Medtronic, Minneapolis, MN, USA) was secured in the aortic root for antegrade coronary perfusion. A 20Fr malleable venous drainage cannula (Medtronic Inc., Dublin, Ireland) was placed into the left atrium (LA) for right atrial perfusion as well as an 8Fr-angled cannula for LA pressure monitoring. All the remaining branches were ligated. The heart and cannulae were de-aired and connected to the perfusion apparatus (Figure 1C).

### End of perfusion

Experimental data were collected for up to 24 h or until end criteria were met. The end criteria were defined as follows: (1) asystole or intractable arrhythmia; (2) LV systolic pressure consistently <30 mmHg for piglet heart experiments and <50% of NEHP baseline for juvenile pig heart experiments; or (3) lactate >7 mmol/L on two consecutive assays separated by 1 h (1). We used all three of these parameters as a benchmark for viability in addition to overall visual appearance of the heart and its contractility. We were required to alter our LV systolic pressure cutoff as we had lower baseline LV pressure readings in the adult heart studies. At the end of 24 h, the hearts were decannulated, drained, weighed, and sent to pathology in formalin.

### Tissue analysis

The hearts were weighed immediately after procurement before *ex situ* perfusion and again immediately following the end of perfusion. The weights were compared and data presented as percent weight change. Sections from each cardiac chamber were sampled, weighed (wet weight), and stored in a desiccator for 7 days. These tissue samples were then weighed (dry weight) and the ratio of the wet weight to the dry weight was calculated (wet–dry ratio).

The hearts were sent to pathology for routine hematoxylin and eosin staining. The samples were examined and scored by a veterinary pathologist using a previously described myocardial injury scoring system (1–3). Injury scores ranged from 0 to 3 based on myofiber degeneration, myocardial hemorrhage, interstitial edema, and endothelial changes, with 0 representing no damage and 3 denoting severe. Average scores using the ordinal data for each injury type were reported for each cardiac chamber as well as a combined average for each heart.

### Data collection

The primary goal of this study is to evaluate heart function for a period of 24 h using the modified extracorporeal circuit. During each experimental run, hemodynamic parameters including heart rate, aortic flow, pulmonary artery flow, aortic root, and left ventricular and left atrial pressures were continuously monitored and recorded every 30 min. In addition, blood gases, electrolyte panels, and lactate levels were recorded on an hourly basis (Radiometer A/S, Copenhagen NV, Denmark) with electrolyte replacement as needed. Coronary vascular resistance (CVR, mmHg/min/mL) was calculated as a measurement of mean aortic root pressure (mmHg) divided by PA flow (mL/min). Cardiac function was assessed using LV systolic pressures, LA pressures*,* coronary vascular resistance, and lactate levels. Oxygen content and glucose concentration were measured in the infusion (i.e., AO) and drainage (i.e., PA) blood, and oxygen kinetics (consumption and extraction) were calculated hourly.

CVR was calculated as follows:

$$\mathrm{CVR}\left(\frac{\mathrm{mmHg}}{\mathrm{mL}/\min}\right)=\frac{\mathrm{Aortic}\;\mathrm{root}\;\mathrm{mean}\;\mathrm{arterial}\;\mathrm{pressure}\;(\mathrm{mmHg})}{\mathrm{Pulmonary}\;\mathrm{artery}\;\mathrm{blood}\;\mathrm{flow}\;(\mathrm{mL}/\min)\;}$$

Oxygen consumption was calculated as follows:

$$\begin{array}{rl} & O_{2}\;\mathrm{consumption}\left(\frac{\mathrm{mL}}{\min}\right)=(\mathrm{Arterial}\;O_{2}\;\mathrm{Content}-\\ & \;\mathrm{Venous}\;O_{2}\;\mathrm{Content})\times \mathrm{Pulmonary}\;\mathrm{artery}\;\mathrm{blood}\;\mathrm{flow} \end{array}$$

### Statistical analysis

Continuous variables are reported as mean ± standard error. Comparisons between continuous variables were conducted using Student's *t*-test. Heart survival rates were calculated using the Kaplan–Meier method. *p*-values less than 0.05 were considered statistically significant. A multiple comparison test was performed using GraphPad Prism version 10.0.0 for Windows (GraphPad Software, Boston, MA, USA; www.graphpad.com).

## Results

**Figure 2:**
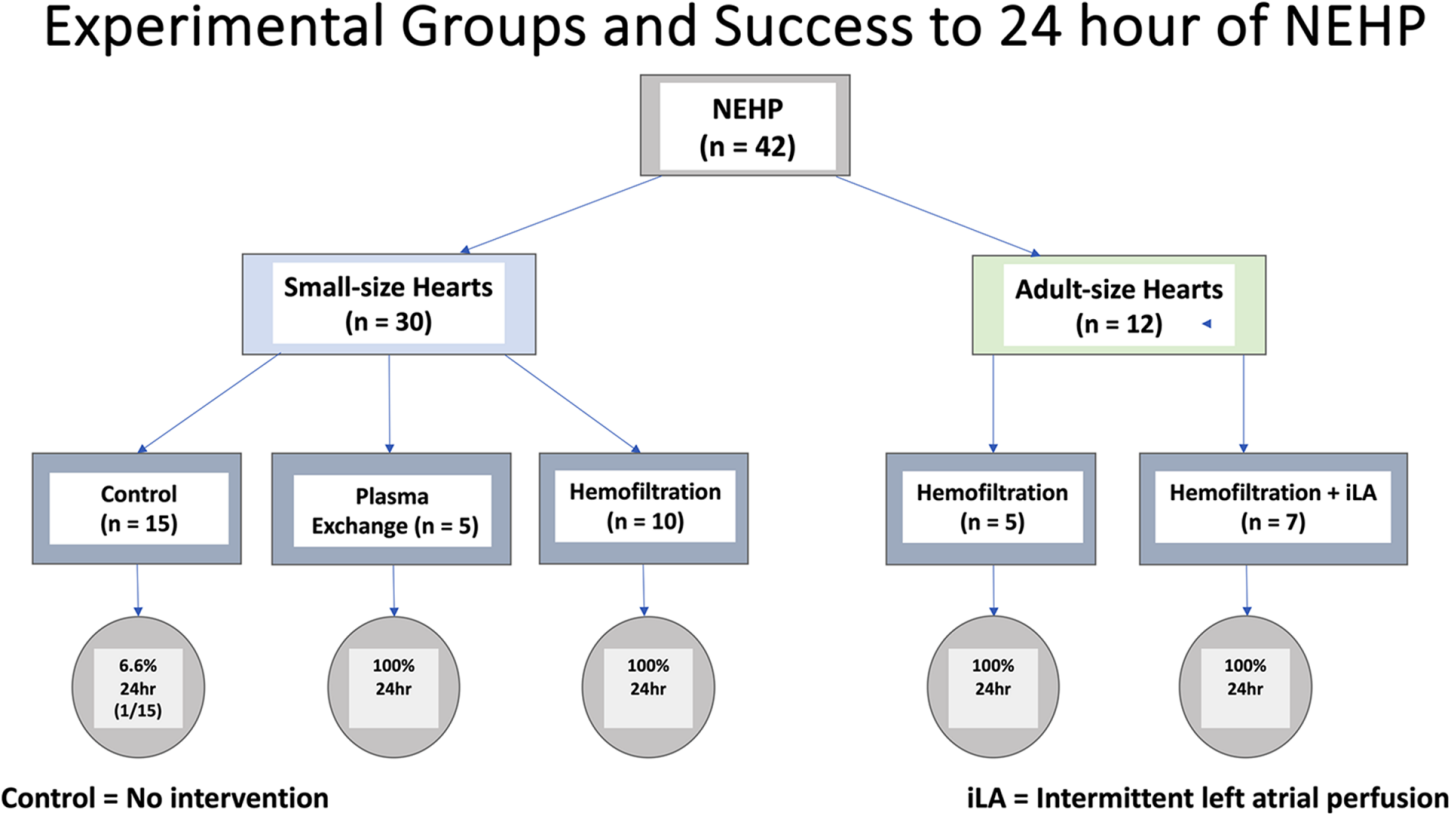
A flow chart summarizing experimental groups and survival to 24 h of NEHP.

An NEHP system was developed for Langendorff perfusion using a blood-derived perfusate (leukocyte/thrombocyte depleted blood). Of the 42 animals, 27 were part of intervention groups including plasma exchange or hemofiltration experiments and 15 were control animals. All 27 hearts in the experimental groups (plasma exchange and hemofiltration) survived up to 24 h. Fourteen (93.3%) control hearts failed between 10 and 21 h and one control heart (6.6%) lasted 24 h (Figure 2).

### Pediatric model with plasma exchange

In the plasma exchange experiments, 10 piglet hearts from animals weighing a median 9 kg (8–10 kg) were utilized. Five separate piglet hearts were perfused for 24 h, maintaining physiologic rhythms, contractility, and response to epinephrine challenge, prior to elective termination. An additional five controls were perfused without plasma exchange, none of which were successful through 24 h (failure at 15, 16, 17, 17, and 24 h).

**Figure 3:**
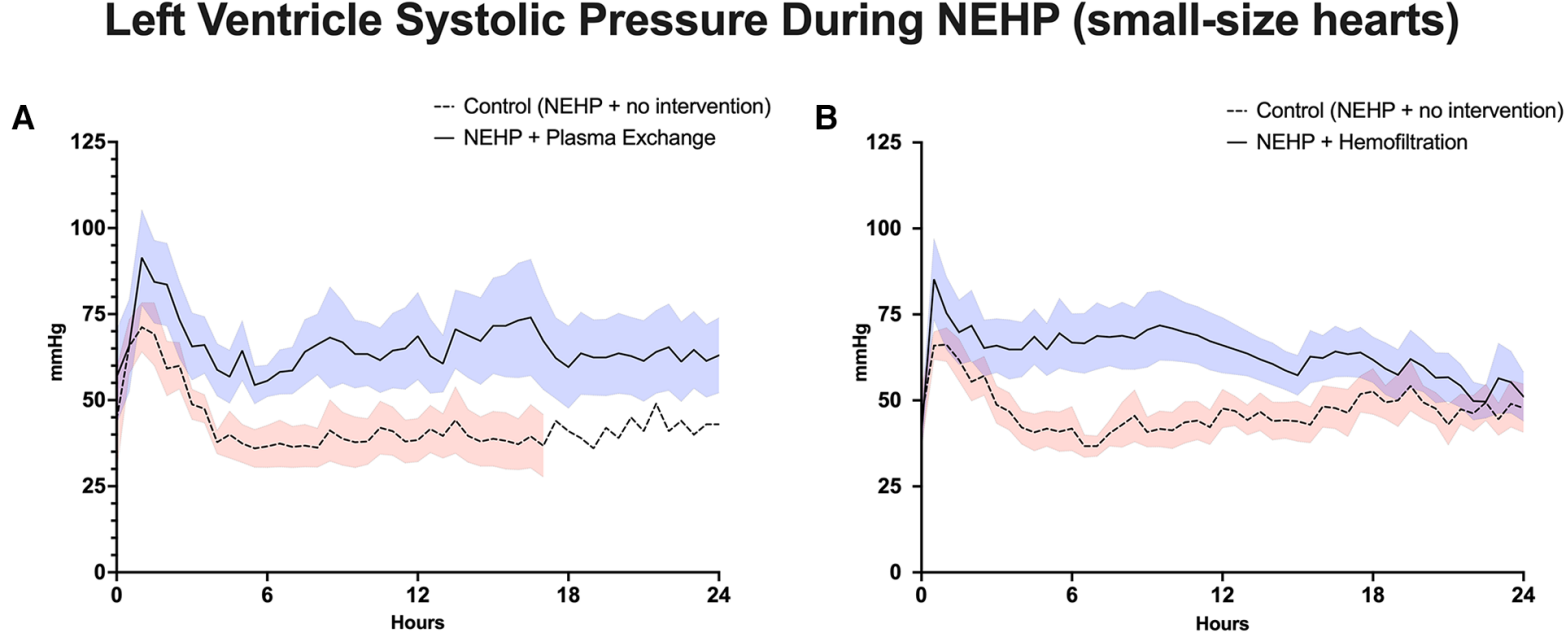
Left ventricle systolic pressure during NEHP. (**A**) Pediatric hearts with plasma exchange; (**B**) pediatric hearts with hemofiltration.

**Figure 4:**
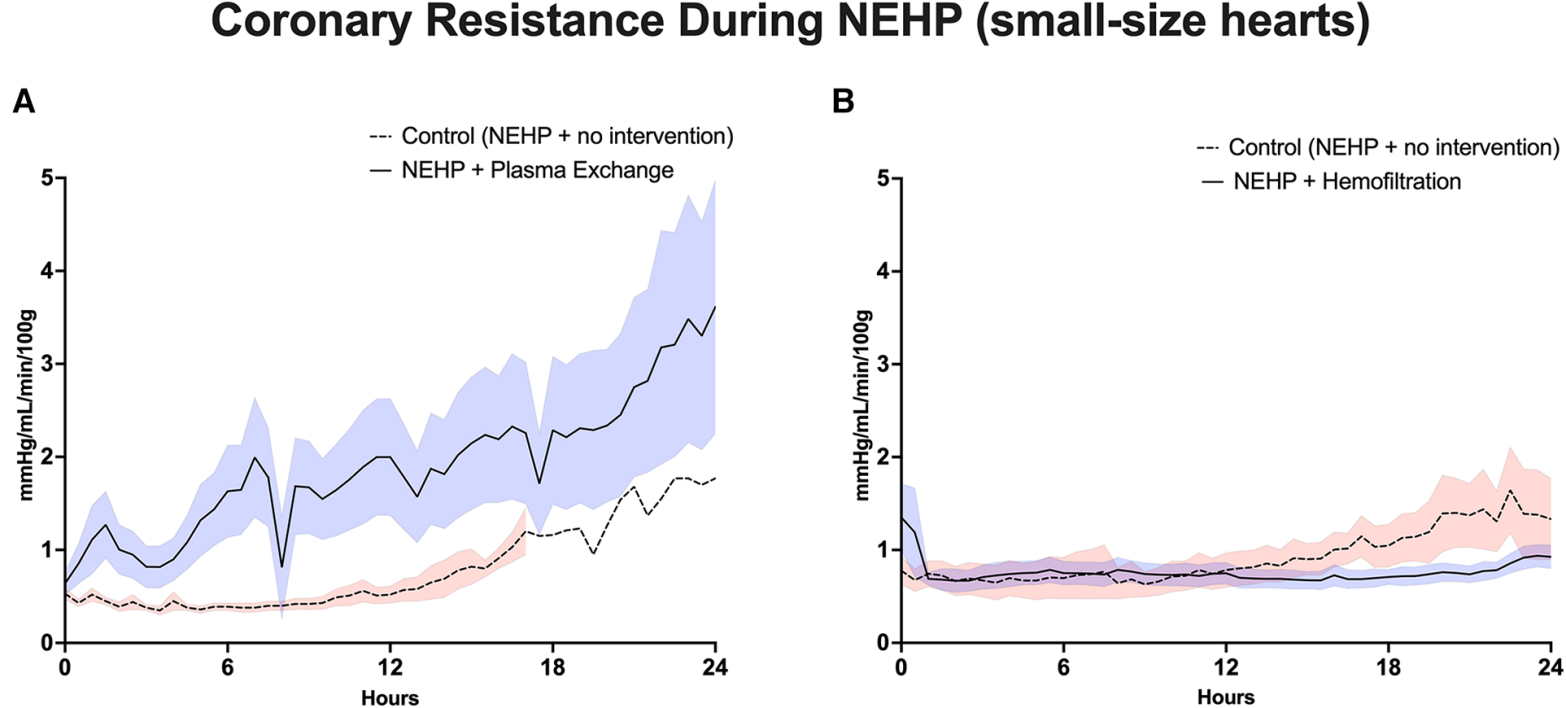
Coronary vascular resistance during NEHP. (**A**) Pediatric hearts with plasma exchange; (**B**) pediatric hearts with hemofiltration.

**Figure 5:**
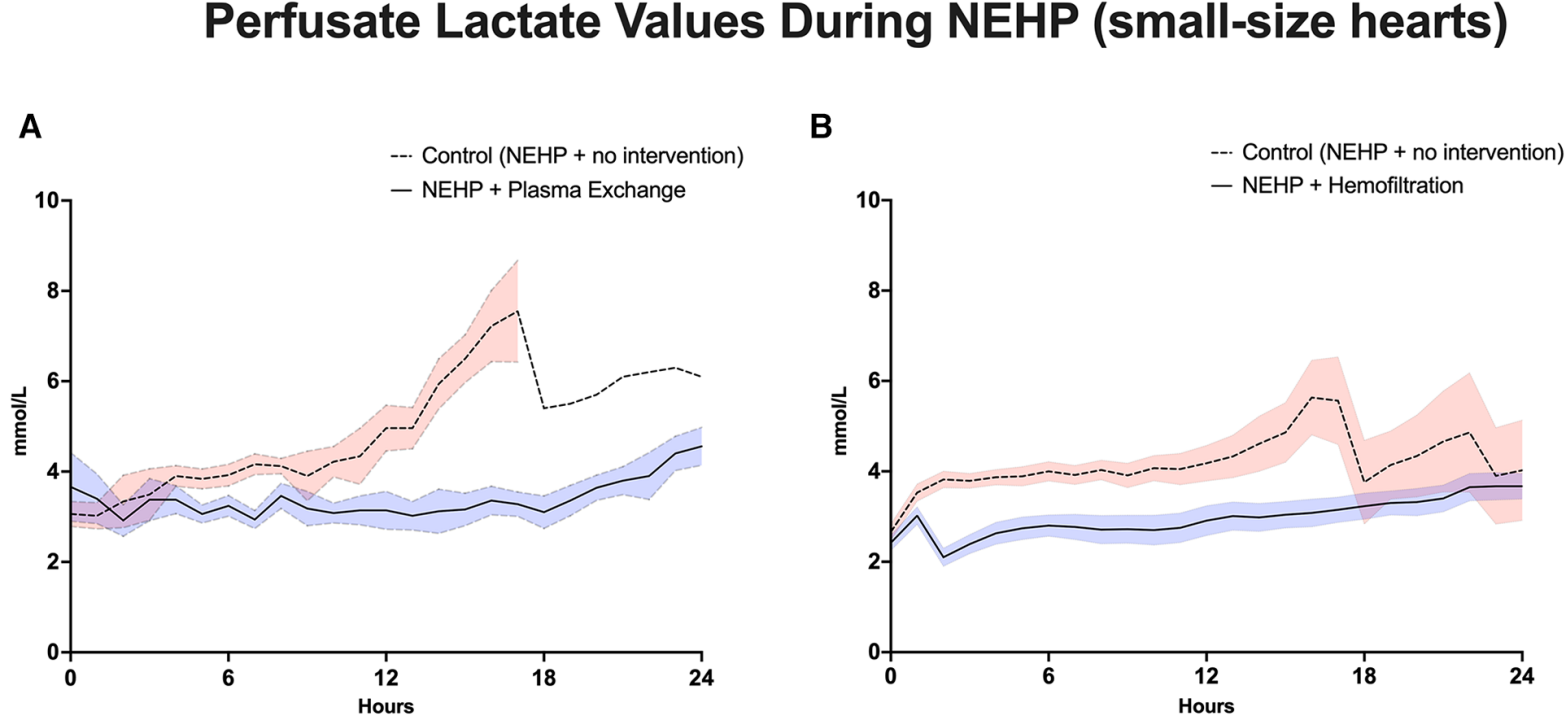
Lactate values during NEHP. (**A**) Pediatric hearts with plasma exchange; (**B**) pediatric hearts with hemofiltration.

Plasma exchange hearts maintained higher, although not statistically significant, LV systolic pressures at the end of perfusion compared with controls (63 ± 10.9 vs. 37 ± 22.0 mmHg, *p* > 0.1). However, this trend was seen throughout the life of the prep as the plasma exchange hearts were able to recover to 80% of baseline LV systolic pressure compared with 50%–60% of baseline for the control hearts (Figure 3A). Coronary resistance was on average similar for the plasma exchange hearts (1.39 ± 0.36 vs. 01.46 ± 0.79 mmHg/mL/min per 100 g of cardiac tissue, *p* > 0.05). However, in the control group, only one heart lasted 24 h, which increased during the life of the experiment; otherwise, the control group had lower coronary resistance values (Figure 4A). Cardiac metabolism demonstrated significantly higher lactate levels for the control hearts (3.6–7.6 vs. 2.8–4.2 mmol/L), which was statistically significant for most of the experiments (Figure 5A). Increased oxygen metabolism was seen in the plasma exchange hearts (2.89 ± 0.1 vs. 1.8 ± 0.1 mL/min/100 g, *p* < 0.05), compared with the control ones.

The final rate of weight change (from start to end of perfusion) was <2% in the plasma group and >50% in the control group, *p* ≤ 0.005. The wet–dry ratio of the plasma exchange piglet hearts was similar between both groups’ LV (5.2 ± 0.2 vs. 5.1 ± 0.2) and RV (5.0 ± 0.5 vs. 4.7 ± 0.3).

### Pediatric model with hemofiltration

In the pediatric hemofiltration experiments, there were 28 piglet hearts from animals with an average weight of 8 kg (6–10 kg). Ten hearts received NEHP with hemofiltration and 10 controls were perfused with NEHP alone. Every hemofiltration-treated heart maintained viability at 24 h and the experiment was electively terminated. Only four control hearts were considered viable at 24 h (two failed at 16 h, three at 17 h, and one at 21 h).

LV systolic pressures were significantly higher in the hemofiltration group than in the control group at the 24-h mark (53.5 ± 6.21 vs. 36.3 ± 4.58 mmHg, *p *< 0.05). In addition, the hemofiltration hearts maintained a statistically equivalent LV systolic pressure from start to end of the experiment (71.7 ± 10.30 to 53.5 ± 6.21 mmHg, *p* < 0.21), while the control hearts saw a decreased pressure over the life of the experiment (55.4 ± 5.62 to 36.3 ± 4.58 mmHg, *p* < 0.01) (Figure 3B). Coronary resistance was maintained from baseline through the end of perfusion in hemofiltration experiments (0.70 ± 0.14 to 0.83 ± 0.11 mmHg/mL/min/100 g of cardiac tissue, *p* > 0.05). In contrast, the coronary resistance doubled from baseline to end of experiment in the control hearts (0.66 ± 0.15 to 1.32 ± 17 mmHg/mL/min/100 g of cardiac tissue, *p* < 0.01) (Figure 4B). At perfusion end, the hemofiltration hearts had a significantly lower coronary resistance than control hearts (*p *< 0.05). Throughout the perfusion period, lactate levels in the hemofiltration hearts were consistently lower in comparison with those in the control group (*p* < 0.01). At the termination of perfusion, lactate levels were measured at 3.67 ± 0.27 mmol/L in the hemofiltration group and 6.20 ± 0.84 mmol/L in the control group (*p* < 0.01) (Figure 5B). Although the control hearts saw a dip in total oxygen consumption from baseline to end of perfusion (0.030 ± 0.005 to 0.018 ± 0.003 mL/min/g tissue, *p *= 0.05), there was no difference between hemofiltration and control hearts.

The final rate of weight change (start to end of perfusion) was <2% in the hemofiltration group and 8%–12% in the control group. The wet–dry ratio of piglet hearts in the hemofiltration group was lower than that of control hearts for both the LV (3.9 ± 0.5 vs. 6.1 ± 0.7, *p* = 0.024) and the RV (4.5 ± 0.6 vs. 5.7 ± 0.4, *p* = 0.048).

### An adult-size heart model with hemofiltration

A scientific and translational critique of the piglet (small-size) hearts experiments was the applicability of the results to a larger model. In response, the subsequent two experiments were devised with juvenile pigs to represent an adult model using a second-generation perfusion circuit (Figure 1B). The following data are unpublished and currently under review.

The first experiment successfully perfused five consecutive juvenile pigs, with an average weight of 48.2 ± 3 kg for 24 h. Contractility of the heart was maintained throughout perfusion with a baseline average of 36.6 ± 7.9 mmHg compared with 27 ± 5.5 mmHg at perfusion end (24 h). Similarly, coronary resistance was preserved from baseline 0.79 ± 0.10 mmHg/L/min through the end of perfusion 0.93 ± 0.28 mmHg/L/min. The lactate average at 24 h was 2.6 ± 0.3 mmol/L.

The final rate of weight change (start to end of perfusion) was <2%.

### iLA perfusion

The following experiment maintained Langendorff perfusion with intermittent 30-min episodes of left atrial (iLA) perfusion every 4–6 h in seven (*n* = 7) hearts (Figure 1C). Otherwise, the same experimental parameters were maintained.

**Figure 6:**
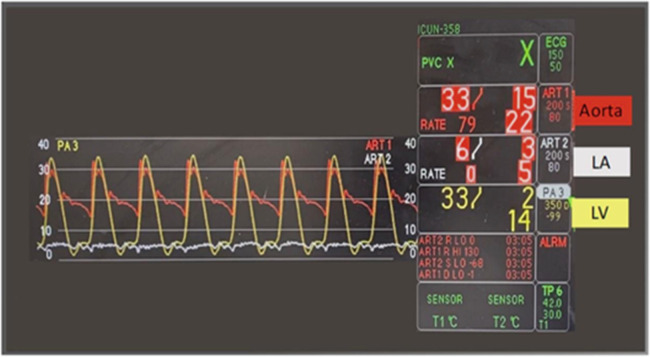
Pressure waveform during iLA perfusion low-flow working mode.

Mean hemodynamic pressures were continuously monitored with an LV systolic pressure of 18.0 ± 6.3 mmHg, an LA pressure of 5.6 ± 2.6 mmHg, and a calculated coronary vascular resistance of 170 ± 58 mmHg/mL/min. During iLA assessment of the cardiac function, no differences were observed throughout the perfusion process during the 30-min iLA tests at low flows. During iLA, cardiac pressures and waveforms were measured and assessed (Figure 6).

The final rate of weight change (start to end of perfusion) was 8%–10%.

### Histopathology and tissue analysis

**Figure 7:**
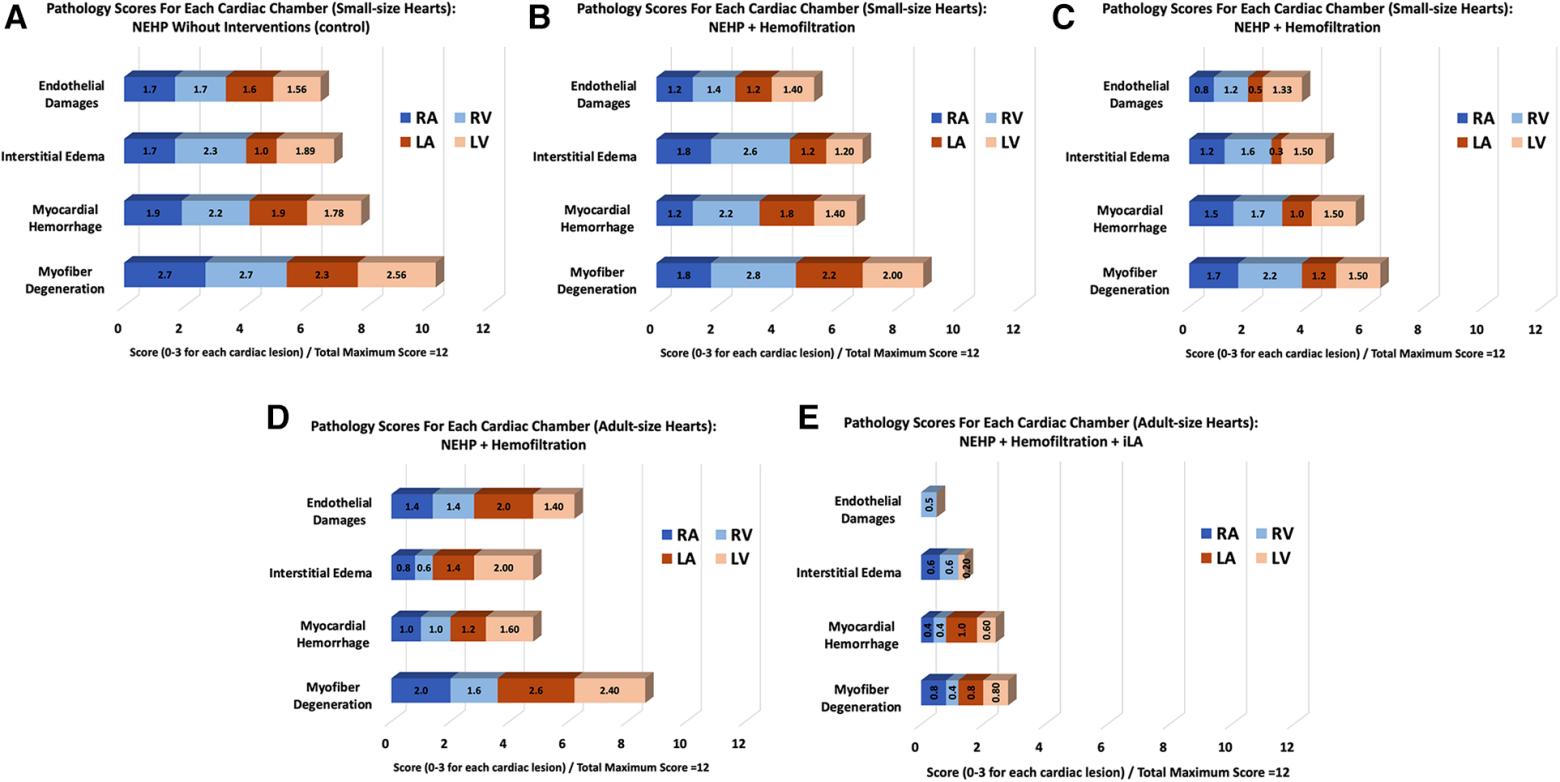
A summary of pathological scores for each experimental group. Pediatric (small-size) hearts: (**A**) Control, (**B**) NEHP + plasma exchange,, and (**C**) NEHP + hemofiltration; Adult-size hearts: (**D**) NEHP + hemofiltration and (**E**) NEHP + hemofiltration + iLA.

The pathology scores for each cardiac chamber in the hearts of the control group were higher than those of all experimental groups (Figure 7A). All experiments in which the hemofilter was used throughout the NEHP period demonstrated lower interstitial edema and endothelial changes for all chambers of the heart (Figures 7B–E). The hearts in the iLA group had significantly lower scores for all chambers of the heart when compared with those in all other groups (Figure 7E). The scores in the adult-size hearts after NEHP + hemofiltration had a higher value (not significant) compared with the results of the pediatric hearts because of positive contamination of bacteria in the samples. This was due to the fact that biofilm formation was observed in reused plastic components of the NEHP system.

## Discussion

This manuscript details experiments performed in our laboratory using NEHP for 24 h, based on our previous successful 3-day perfusion with plasma exchange with paracorporeal animal experiments. The reported experiments were performed in both piglet (small-size) and juvenile pig (adult-size) hearts with varying interventions. Both hemofiltration and plasma exchange groups for piglet hearts performed better than control hearts (no interventions), consistently demonstrating adequate perfusion parameters for the duration of the experiment. The juvenile pig hemofiltered hearts also lasted 24 h and had stable parameters. In our iLA perfusion prep of adult-size hearts, we were able to adequately assess heart function using this model for 24 h and continued to demonstrate adequate heart perfusion and function for the duration of the experiment. These experiments add to the current extended perfusion literature in heart perfusion as well as other organs and demonstrate the feasibility of our circuit and experimental model.

Normothermic *ex situ* heart perfusion has been utilized since 1970, when isolated canine hearts were blood-perfused retrogradely through the aorta in order to evaluate left ventricular performance (4). More recently, studies worked to extend the preservation time with NEHP. Trahanas et al. were able to achieve 12 h of normothermic *ex situ* heart perfusion by perfusing the heart with platelet- and leukocyte-reduced blood supplemented with dextran 40, cell culture media, insulin, and antibiotics with perfusate exchange every 2 h. The successful hearts had lower potassium, lactate, percent weight gain, and pathological injury scores than the hearts that did not achieve 12 h of perfusion (5). Then, McLeod et al. demonstrated that normothermic *ex situ* heart perfusion for up to 72 h using cross-plasma circulation (XC-plasma) from a live, awake paracorporeal sheep was feasible (1). In this study, six ovine hearts were perfused for 72 h using plasma cross-circulation at a rate of 1 L/min with a live, awake paracorporeal sheep. Controls were seven perfused hearts without cross-circulation. Experiments were electively ended at 72 h, and epinephrine (0.1 mg) was delivered to demonstrate hormonal responsiveness. All controls failed at 6–1 h. All six hearts perfused for 72 h maintained normal heart function, metabolism, and responsiveness to epinephrine. Blood gases, electrolytes, and lactate levels were normal and stable throughout the study. From this experience, our group has focused on preserving hearts for 24 h or longer by not only providing warm oxygenated blood, but also filtrating the perfusate and adding nutrition during NEHP.

Clinically, the only commercially available normothermic *ex situ* heart perfusion device is the OCS Heart (Transmedics, Andover, MA, USA). Transplanted hearts that utilized the OCS Heart were shown to have superior 1- and 2-year survival rates, less primary graft failure, less severe acute rejection, and less acute renal failure than transplant hearts preserved with cold Custodiol (6). Patients who received an OCS Heart–perfused heart transplant had comparable 30-day survival rates (7), 2-year survival rates, freedom from cardiac allograft vasculopathy, non-fatal major cardiac events, and biopsy-proven cellular rejection or antibody-mediated rejection to cold storage–preserved hearts (8). In transplants in which a long total ischemic time was expected, patients with OCS Heart–perfused hearts had 30-day and 6-month survival rates of 94.7% and 88%, respectively, and severe LV or RV primary graft dysfunction (PGD) rates of 10.7% (9).

A few cases of successful heart transplantation in humans after extended normothermic perfusion using the OCS system have been reported. The first case was reported by Stamp et al. (10), in which a heart was preserved for 8.5 h with successful transplantation into a 39-year-old recipient. In this case, extracorporeal membrane oxygenation (ECMO) was used immediately after transplant for 17.5 h, and the recipient was extubated after 72 h and discharged after 15 days. The second report with successful human transplantation was published by Kaliyev et al. (11), where hemofiltration was added to the OCS system to preserve a heart for 16 h. The graft was successfully transplanted to a 48 year-old recipient. Similar to the report by Stamp *et al*., in this second case of prolonged OCS preservation, ECMO was required for 44 h after transplant, and the patient was extubated at 72 h and discharged home after 24 days with normal biventricular function.

These reports corroborate our results from animal data (pig and sheep) and prove the fundamental principle that normothermic perfusion with the addition of a hemofilter can support organs for prolonged periods of time. Further, both groups concluded that despite optimal perfusion parameters and lactate levels, there was PGD and ECMO was required to support recipients immediately after transplant. This is one of our main conclusions, that current biomarkers and parameters used to assess organ viability lack the objectivity to assess organ function as electrolytes and products of cellular metabolism are removed during hemofiltration. Currently, OCS cannot provide direct measurements of donor heart hemodynamic function. Our perfusion apparatus and perfusion protocols allowed for the monitoring of physiological parameters in a working heart, as we discussed in the iLA studies, in a low-flow environment. Other groups have reported the use of pressure–volume loops and surface echocardiography as methods to assess heart function during NEHP (12). iLA also provides a “working” heart mode setting where echocardiography and pressure/volume curves can be used to not only assess the function of hearts but also to monitor the effects of different interventions to improve graft viability during NEHP. In addition, perfusing hearts in working mode has shown some benefits by mitigating oxidative stress, as reported by Dr. Freed's group (13), and myocardial injury, as demonstrated by the pathology in the iLA group. Working mode NEHP has its limitations, as reported by Olkowicz et al. (14). This group reported the relationship between dysregulation of the cardiac metabolome and declining myocardial function during 8 h of NEHP using the STEEN solution™ and red blood cells. They performed physiological measurements by loading the left ventricle to a pressure of 8 mmHg three times during NEHP. In addition, they demonstrated that several metabolic pathways are altered during NEHP, with emphasis on increased inflammatory and oxidative stress response and compromised substrate utilization.

There are lessons from prolonged perfusion of other organs that may impact NEHP. Chapman et al. perfused bovine livers with normothermic blood for 24 h (15). Vogel et al. transplanted porcine livers after perfusing with blood for 48 h. They showed that the livers sustained bile production and metabolic activity for 5 days and there was a 100% survival rate (16). Another focus of *ex situ* liver perfusion studies is the rehabilitation of damaged organs. Schön et al. showed that 4 h of normothermic perfusion was sufficient to recover liver function after an hour of warm ischemia (17). St Peter et al. perfused porcine livers for 24 h after an hour of warm ischemia and the perfused livers demonstrated superior synthetic function, substrate utilization, and perfusion hemodynamics as well as less cellular injury compared with livers preserved with cold storage after warm ischemia (18). This rehabilitation can be applied to heart perfusion if we can further extend *ex situ* heart perfusion to cardiac grafts from marginal donors or from donors after circulatory arrest.

*Ex situ* lung perfusion has also shown the ability to maintain lung function and rehabilitate damaged lungs. Steen et al. demonstrated that porcine lungs perfused for 6 h and then transplanted and reperfused for 24 h all maintained baseline blood gas transfer and pulmonary vascular resistance (19). Spratt et al. showed that 24 h of *ex situ* lung perfusion improved hemodynamics and compliance after warm ischemia (20). They then transplanted those lungs, reperfused them for 4 h, and found that lung function in the first 8 h of *ex situ* lung perfusion was able to predict lung function posttransplant (21).

Our work adds to the expanding research on normothermic *ex situ* organ perfusion, specifically on heart resuscitation and function assessment. The addition of iLA perfusion enables real-time objective quantifiable cardiac function assessment during NEHP, a unique feature with significant impact during the assessment of marginal donor hearts and hearts from donors after circulatory death.

## Limitations

This is a translational model of heart preservation in piglets (small-size hearts) and juvenile pigs (adult-size hearts). The animal model does not simulate the scenario of human heart donation (brain death or cardiopulmonary death physiology) as the animals are young and healthy. In addition, the use of blood products from the blood bank showed negative effects during NEHP, as reported by Chew et al. (22). However, this was a single case report that did not account for the effects of the citrate-based anticoagulant and the age of the blood. Another limitation of our results is related to contamination with bacteria of the histology from the adult-size studies in the NEHP + hemofiltration series. This is due to the fact that some of the NEHP system components were reused during the early studies with juvenile pigs, and we observed a contamination of bacteria and biofilm formation in some plastic components that affected the histological scores.

## Conclusion

Prolonged (24 h or more) heart preservation is feasible with our NEHP perfusion technique. LA perfusion, even in coronary flow (working) mode, enables real-time functional assessment during NEHP. To increase the preservation period beyond 24 h, infection control and nutritional support need to be optimized. The current work proves the concept in a large animal model that NEHP has the potential to increase the organ pool by (1) increasing the possible donor/recipient distance; (2) enabling an objective assessment of heart function with the addition of working mode perfusion; (3) considering previously discarded hearts for transplantation; and (4) developing heart-donor-type–specific therapies during NEHP. Further studies that include working mode NEHP, objective parameters to assess heart function, and the development of novel biomarkers to assess heart viability are required to translate prolonged NEHP successfully and routinely into clinical practice.

## Data Availability

The raw data supporting the conclusions of this article will be made available by the authors, without undue reservation.

## References

[1] McLeod JS Poling C Church JT Jung J Sarosi E Langley M Ex vivo heart perfusion for 72 hours using plasma cross circulation. ASAIO J. (2020) 66(7):753–91. 10.1097/MAT.0000000000001061 31453833

[2] Tchouta L Drake D Hoenerhoff M Rojas-Pena A Haft J Owens G Twenty-four-hour normothermic perfusion of isolated ex vivo hearts using plasma exchange. J Thorac Cardiovasc Surg. (2022) 164(1):128–38. 10.1016/j.jtcvs.2020.11.158 33485659

[3] Johnson MD Fallon BP Langley M Kayden A Shenton H Schneider B Prolonged (24-hour) normothermic ex vivo heart perfusion facilitated by perfusate hemofiltration. ASAIO J. (2022) 68(10):1282–9. 10.1097/MAT.0000000000001649 36194099

[4] Monroe RG Gamble WJ LaFarge CG Kumar AE Manasek FJ. Left ventricular performance at high end-diastolic pressures in isolated, perfused dog hearts. Circ Res. (1970) 26(1):85–99. 10.1161/01.RES.26.1.85 5410095

[5] Trahanas JM Witer LJ Alghanem F Bryner BS Iyengar A Hirschl JR Achieving 12 hour normothermic ex situ heart perfusion: an experience of 40 porcine hearts. ASAIO J. (2016) 62(4):470–6. 10.1097/MAT.0000000000000382 27164040 PMC4925299

[6] Koerner MM Ghodsizad A Schulz U El Banayosy A Koerfer R Tenderich G. Normothermic ex vivo allograft blood perfusion in clinical heart transplantation. Heart Surg Forum. (2014) 17(3):E141–145. 10.1532/HSF98.2014332 25002389

[7] Ardehali A Esmailian F Deng M Soltesz E Hsich E Naka Y Ex-vivo perfusion of donor hearts for human heart transplantation (PROCEED II): a prospective, open-label, multicentre, randomised non-inferiority trial. Lancet. (2015) 385(9987):2577–84. 10.1016/S0140-6736(15)60261-6 25888086

[8] Chan JL Kobashigawa JA Reich HJ Ramzy D Thottam MM Yu Z Intermediate outcomes with ex-vivo allograft perfusion for heart transplantation. J Heart Lung Transplant. (2017) 36(3):258–63. 10.1016/j.healun.2016.08.015 27646064

[9] Schroder JN D’Alessandro D Esmailian F Boeve T Tang P Liao K Successful utilization of extended criteria donor (ECD) hearts for transplantation—results of the OCS™ heart EXPAND trial to evaluate the effectiveness and safety of the OCS heart system to preserve and assess ECD hearts for transplantation. J Heart Lung Transplant. (2019) 38(4):S42. 10.1016/j.healun.2019.01.088

[10] Stamp NL Shah A Vincent V Wright B Wood C Pavey W Successful heart transplant after ten hours out-of-body time using the TransMedics organ care system. Heart Lung Circ. (2015) 24(6):611–3. 10.1016/j.hlc.2015.01.005 25697385

[11] Kaliyev R Bekbossynov S Nurmykhametova Z. Sixteen-hour ex vivo donor heart perfusion during long-distance transportation for heart transplantation. Artif Organs. (2019) 43(3):319–20. 10.1111/aor.13359 30585343 PMC6590649

[12] Dang Van S Brunet D Akamkam A Decante B Guihaire J. Functional assessment of the donor heart during ex situ perfusion: insights from pressure-volume loops and surface echocardiography. J Vis Exp. (2022) (188):e63945. 10.3791/63945 36314840

[13] Hatami S Qi X Khan M Forgie K Himmat S Tkachuk B Oxidative stress and related metabolic alterations are induced in ex situ perfusion of donated hearts regardless of the ventricular load or leukocyte depletion. Am J Transplant. (2023) 23(4):475–83. 10.1016/j.ajt.2022.11.027 36695686

[14] Olkowicz M Ribeiro RVP Yu F Alvarez JS Xin L Yu M Dynamic metabolic changes during prolonged ex situ heart perfusion are associated with myocardial functional decline. Front Immunol. (2022) 13:859506. 10.3389/fimmu.2022.859506 35812438 PMC9267769

[15] Chapman ND Goldsworthy PD Volwiler W Nyhus LM Martinis AJ. The isolated perfused bovine liver. J Exp Med. (1961) 113(6):981–96. 10.1084/jem.113.6.981 13692411 PMC2137432

[16] Vogel T Brockmann JG Pigott D Neil DAH Muthusamy ASR Coussios CC Successful transplantation of porcine liver grafts following 48-hour normothermic preservation. PLoS One. (2017) 12(11):e0188494. 10.1371/journal.pone.0188494 29176869 PMC5703476

[17] Schön MR Kollmar O Wolf S Schrem H Matthes M Akkoc N Liver transplantation after organ preservation with normothermic extracorporeal perfusion. Ann Surg. (2001) 233(1):114–23. 10.1097/00000658-200101000-00017 11141233 PMC1421174

[18] St Peter SD Imber CJ Lopez I Hughes D Friend PJ. Extended preservation of non-heart-beating donor livers with normothermic machine perfusion. Br J Surg. (2002) 89(5):609–16. 10.1046/j.1365-2168.2002.02052.x 11972552

[19] Steen S Liao Q Wierup PN Bolys R Pierre L Sjöberg T. Transplantation of lungs from non-heart-beating donors after functional assessment ex vivo. Ann Thorac Surg. (2003) 76(1):244–52; discussion 252. 10.1016/S0003-4975(03)00191-7 12842550

[20] Spratt JR Mattison LM Iaizzo PA Brown RZ Helms H Iles TL An experimental study of the recovery of injured porcine lungs with prolonged normothermic cellular ex vivo lung perfusion following donation after circulatory death. Transpl Int. (2017) 30(9):932–44. 10.1111/tri.12981 28493634

[21] Spratt JR Mattison LM Iaizzo PA Meyer C Brown RZ Iles T Lung transplant after prolonged ex vivo lung perfusion: predictors of allograft function in swine. Transpl Int. (2018) 31(12):1405–17. 10.1111/tri.13315 29981183

[22] Chew HC Scheuer S Dhital K Macdonald P. Banked blood for normothermic machine perfusion of the donor heart: a clinical perspective. J Heart Lung Transplant. (2019) 38(12):1322. 10.1016/j.healun.2019.09.001 31606296

